# SARS-CoV-2 Infection Exacerbates Hypertensive Disorders in Pregnancy Through Vascular and Immune Pathways

**DOI:** 10.3390/ijms27135891

**Published:** 2026-06-30

**Authors:** Marta Fabre, Ana Medel-Martinez, Pilar Calvo, Natalia Abadia-Cuchi, Sara Ruiz-Martinez, Maria Peran, Cristina Paules, Alberto Montolío, Beatriz Jimeno-Beltrán, Javier Godino, Alberto Cebollada-Solanas, Mark Strunk, Fatima Crispi, Daniel Oros, Jon Schoorlemmer

**Affiliations:** 1 Obstetrics Department, Hospital Clínico Universitario Lozano Blesa Zaragoza, 50009 Zaragoza, Spain; 2 Instituto de Investigación Sanitario de Aragón (IIS Aragon), 50009 Zaragoza, Spain; 3 Instituto Aragonés de Ciencias de la Salud (IACS), 50009 Zaragoza, Spainacebolladaso.iacs@aragon.es (A.C.-S.);; 4 BCNatal-Barcelona Center for Maternal-Fetal and Neonatal Medicine (Hospital Clínic and Hospital Sant Joan de Déu), Centre for Biomedical Research on Rare Diseases (CIBER-ER), Universitat de Barcelona, 08007 Barcelona, Spain; 5 Hospital Clinic Barcelona, 08036 Barcelona, Spain; 6 Fundación agencia aragonesa para la investigación y el desarrollo, 50018 Zaragoza, Spain

**Keywords:** preeclampsia, pregnancy, SARS-CoV-2

## Abstract

Background: SARS-CoV-2 infection has been linked to an increased risk of hypertensive disorders during pregnancy, particularly preeclampsia (PE). As both conditions involve vascular and endothelial dysfunction, a mechanistic overlap has been proposed. This study examines the relationship between maternal COVID-19 and preeclampsia by analyzing inflammatory, endothelial, and angiogenic biomarkers in pregnancies with and without these complications. Methods: A case–control study was conducted, including four groups: healthy pregnancies before 2020 (*n* = 10), preeclampsia cases before 2020 (*n* = 10), COVID-19 cases without preeclampsia (*n* = 10), and COVID-19 cases with preeclampsia (*n* = 10). The groups were selected to be comparable in terms of gestational age at blood sampling. Biomarkers related to endothelial, inflammatory, and angiogenic pathways were measured. Results: Significant differences in biomarker levels were detected among the four groups. Regarding endothelial damage, sICAM1 levels were significantly higher in the COVID-PE group compared with the COVID-noPE group (*p* = 0.002). Additionally, vWF (*p* = 0.006), END1 (*p* < 0.001), and sVCAM1 (*p* = 0.030) levels varied significantly across groups. IL8 levels showed significant differences (*p* < 0.001), and were particularly elevated in preeclampsia cases (preCOVID-PE and COVID-PE groups) compared with controls (*p* = 0.005 and *p* < 0.001, respectively). Angiogenic markers sFlt-1, PLGF, and sFlt-1/PLGF exhibited significant group differences (*p* < 0.001). In contrast, maternal SARS-CoV-2 infection in the absence of preeclampsia was not associated with a significant alteration of the sFlt-1/PlGF ratio. Discussion: PE associated with SARS-CoV-2 infection preserved the classical angiogenic signature of preeclampsia, but showed additional endothelial and inflammatory biomarker alterations. These findings support an association between SARS-CoV-2 infection and a distinct endothelial and inflammatory biomarker profile in PE, warranting confirmation in larger prospective studies.

## 1. Introduction

Preeclampsia (PE) is a multisystem, hypertensive pregnancy disorder characterized by new-onset hypertension and proteinuria. PE is a major obstetric complication, affecting approximately 2% to 5% of pregnancies and is associated with an increased risk of preterm birth and fetal intrauterine growth restriction [1]. The etiology of PE remains incompletely understood but it is known that multiple risk factors, including maternal infection, deficient implantation, placental insufficiency and inflammation converge on the presence of uteroplacental ischemia [2]. Multicenter studies with carefully selected control groups have reported an association between SARS-CoV-2 (Severe Acute Respiratory Syndrome Coronavirus 2) infection during pregnancy and an increased risk of developing PE [3,4]. However, the mechanisms linking COVID-19 disease (Coronavirus Disease 2019) and hypertensive disorders of pregnancy (HDP) remain unclear. Our group has previously suggested that SARS-CoV-2 infection during pregnancy may be associated with gestational hypertensive disorders through persistent placental infection and placental damage [5].

However, endothelial damage (ED) is known to be a central feature of PE and COVID-19 disease, contributing to their pathophysiology and serious complications of the disease. Distinctive profiles of circulating biomarkers related to ED have been reported. In PE, increased levels of soluble fms-like tyrosine kinase-1 (sFlt-1) and decreased levels of placental growth factor (PlGF) correlate with the severity of PE, reflecting the impaired angiogenic balance and ED observed in affected pregnancies [6,7,8]. Furthermore, it has been reported that serum levels of soluble cell adhesion molecules (sCAMs) are higher in patients with PE than in healthy pregnant women. On the other hand, endothelial and inflammation biomarkers have been correlated with clinical COVID-19 disease severity [9]. There are close correlations between interleukin 6 (IL-6), fibrinogen and von Willebrand Factor (FvW) and a poor prognosis in patients with SARS-CoV-2 infection [10,11,12]. Finally, several studies discussed the use of biomarkers to distinguish hypertension and endothelial dysfunction caused by COVID-19-related inflammation from preeclampsia. Soldavini et al. concluded [13] that the sFlt-1/PlGF ratio was significantly elevated in non-COVID-19 patients with HDP when compared to both hypertensive and normotensive COVID-19 patients. Palomo et al. [14] demonstrated that both pregnant women with preeclampsia and COVID-19 showed altered profiles of circulating endothelial, inflammatory and angiogenic biomarkers compared to healthy pregnant women. Therefore, even though extensive endothelial dysfunction is common to both PE and COVID-19, their underlying pathophysiological profiles may be different.

This study aimed to better understand the relationship between maternal COVID-19 disease and the increased frequency of PE among pregnant women. We analyzed selected maternal blood markers associated with inflammation, endothelial dysfunction, and angiogenic imbalance in pregnant women during the first year of the pandemic who were diagnosed with COVID-19 or COVID-19 and preeclampsia.

## 2. Results

The demographic and clinical data from the four groups are shown in Table 1. Compared to the other groups, the COVID-PE group demonstrated a markedly higher risk of developing preeclampsia, with elevated risk scores in the first trimester (Risk PE > 100, *p* = 0.026 and Risk PE > 250, *p* = 0.003). This heightened risk was associated with significantly poorer maternal and neonatal outcomes (Table 1).

**Figure 1:**
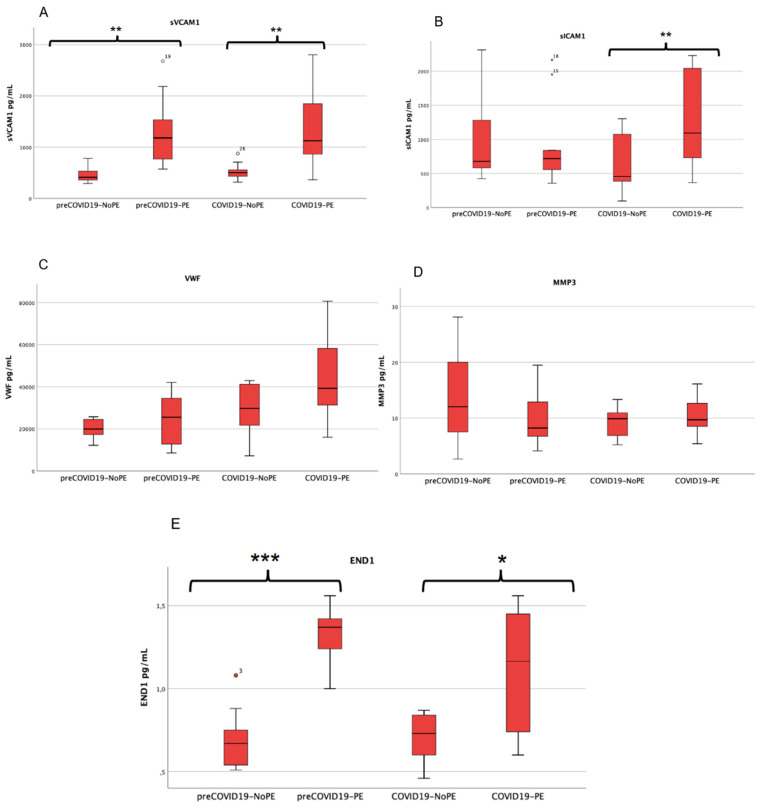
Endothelial damage markers in the four groups. (**A**): Soluble vascular cell adhesion molecule 1 (sVCAM1), (**B**): soluble intercellular adhesion molecule-1 (sICAM1), (**C**): von Willebrand factor (vWF), (**D**): matrix metalloproteinase-3 (MMP3), (**E**): endothelin 1 (EDN1). *p* < 0.05 *; *p* < 0.01 **; *p* < 0.001 ***; circles represent mild outliers (1.5 to 3 times the IQR); asterisks represent extreme outliers (greater than 3 times the IQR). The *Y*-axis for sVCAM1 and sLCAM1 is scaled ×1000.

**Figure 2:**
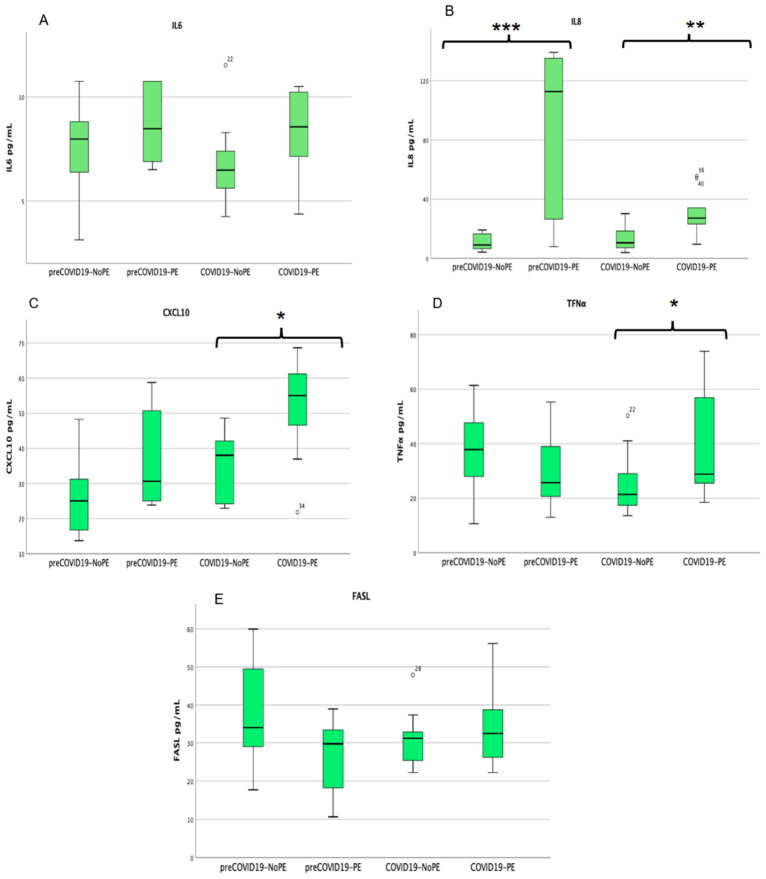
Inflammation markers in the four groups. (**A**): Interleukin 6 (IL6), (**B**): interleukin 8 (IL8), (**C**): C-X-C motif chemokine ligand 10 (CXCL10), (**D**): matrix metalloproteinase-3 (MMP3), (**E**): fas ligand (FASL). *p* < 0.05 *; *p* < 0.01 **; *p* < 0.001 ***; circles represent mild outliers (1.5 to 3 times the IQR); asterisks represent extreme outliers (greater than 3 times the IQR).

**Figure 3:**
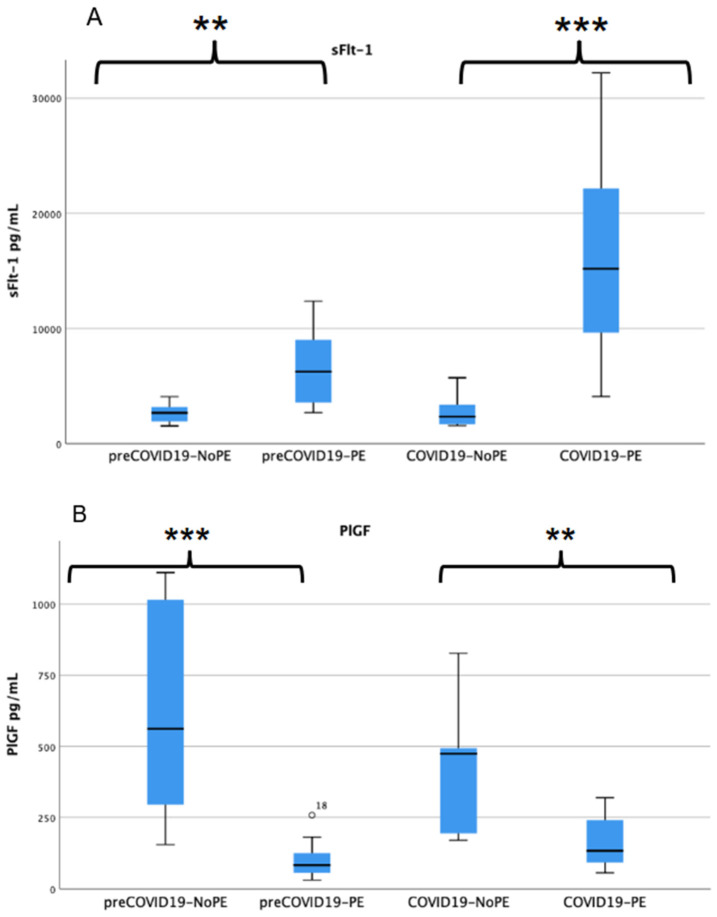

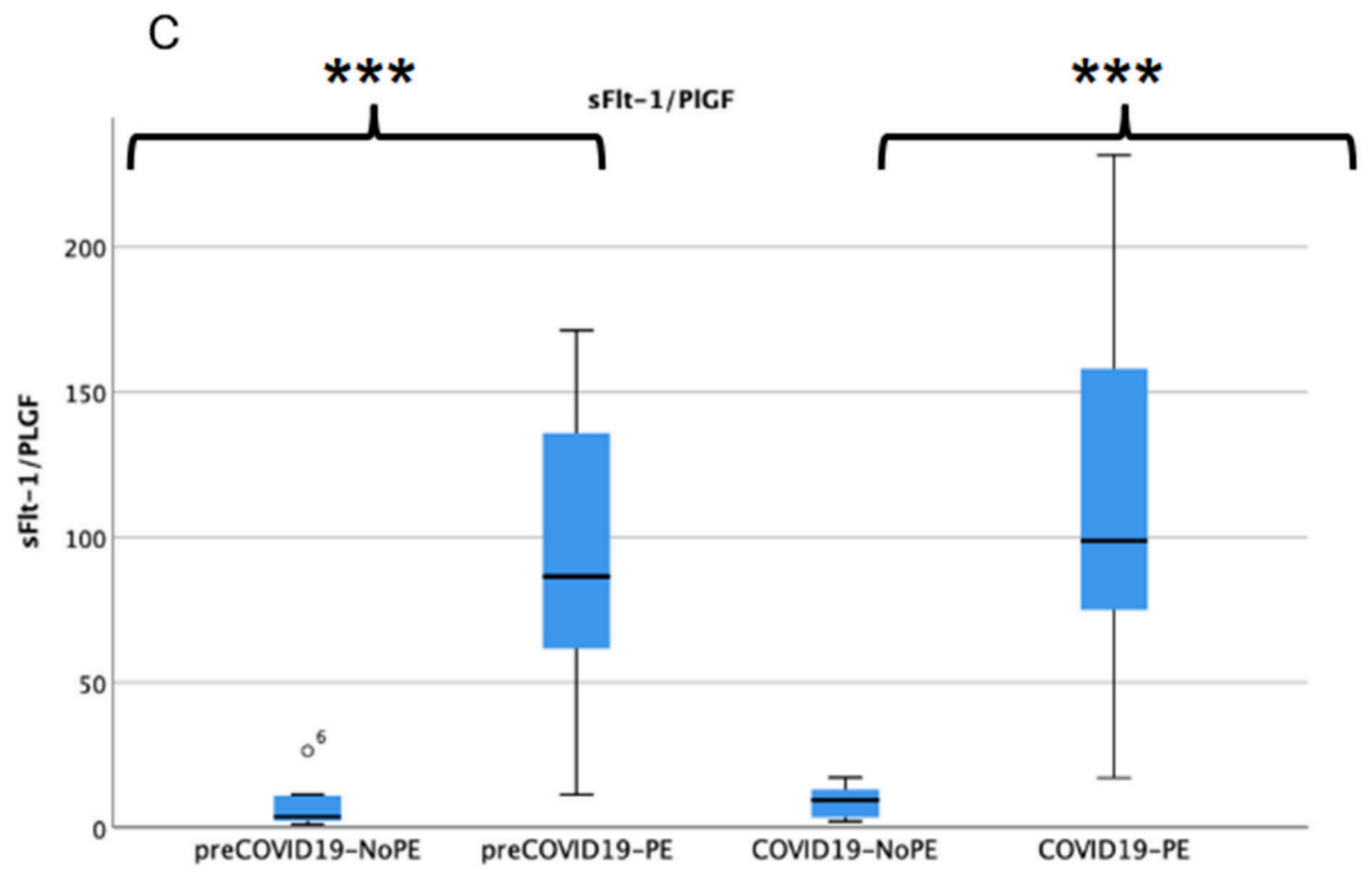
Angiogenic markers in the four groups. (**A**): Soluble fms-like tyrosine kinase-1 (sFLT1), (**B**): placental growth factor (PlGF). (**C**): sFLT1/PlGF ratio. *p* < 0.01 **; *p* < 0.001 ***; circles represent mild outliers (1.5 to 3 times the IQR); asterisks represent extreme outliers (greater than 3 times the IQR).

We analyzed the levels of several biomarkers in maternal blood samples. The results are presented in Table 2. Unadjusted and false discovery rate (FDR)-adjusted *p*-values for pairwise comparisons are provided in Supplementary Table S1. In the pre-pandemic cohort, levels of EDN1, sVCAM1, IL8 (Figure 1 and Figure 2), sFlt-1, PlGF, and the sFlt-1/PlGF ratio (Figure 3) were significantly altered in pregnancies complicated by preeclampsia compared to uneventful pregnancies. After FDR correction, these differences remained significant for EDN1, sVCAM1, IL8, sFlt-1, PlGF, and the sFlt-1/PlGF ratio. When comparing pre-pandemic and pandemic cohorts, these same markers showed very similar levels between the two control groups (preCOVID-NoPE and COVID-NoPE), with no significant differences after FDR correction. Both PE groups, preCOVID-PE and COVID-PE, showed the expected angiogenic profile of preeclampsia, characterized by increased sFlt-1, reduced PlGF, and an elevated sFlt-1/PlGF ratio compared with controls. After FDR correction, sFlt-1, PlGF, and the sFlt-1/PlGF ratio remained significantly altered in the COVID-PE group compared with both preCOVID-NoPE and COVID-NoPE groups. In contrast, SARS-CoV-2 infection in the absence of PE was not associated with a significant change in the sFlt-1/PlGF ratio.

Endothelial damage markers are shown in Figure 1. MMP3 levels were similar across groups. VWF, EDN1, and sVCAM1 showed significant differences in the global analysis. After FDR correction, the main pairwise differences were observed for VWF, sVCAM1, and sICAM1 in the COVID-PE group, while EDN1 and sVCAM1 were increased in PE groups compared with preCOVID-NoPE controls. Inflammatory markers are shown in Figure 2. IL8 and CXCL10 showed significant group differences. After FDR correction, IL8 remained increased in both PE groups compared with preCOVID-NoPE controls, and CXCL10 remained increased in COVID-PE compared with preCOVID-NoPE. IL6, TNF-α, and FASL did not show significant differences after FDR correction.

Finally, angiogenic markers (Figure 3) showed the expected alterations in PE, including elevated sFlt-1, reduced PlGF, and a higher sFlt-1/PlGF ratio (*p* < 0.001 for all comparisons). After FDR correction, these angiogenic differences remained significant for comparisons of both PE groups with preCOVID-NoPE controls and for comparisons of COVID-PE with COVID-NoPE. No significant difference in the sFlt-1/PlGF ratio was observed between preCOVID-NoPE and COVID-NoPE, supporting that COVID-19 in the absence of PE was not associated with the classical angiogenic imbalance of preeclampsia.

## 3. Discussion

To understand the increased incidence of preeclampsia (PE) associated with COVID-19, we evaluated maternal blood biomarkers related to endothelial damage, coagulation, and angiogenesis. Our analysis focused on markers previously linked to PE, using blood samples from study participants divided into four groups: women who delivered either before or during the COVID-19 pandemic, with each group further classified based on SARS-CoV-2 infection status during pregnancy and whether they developed preeclampsia (N = 10 each group). The main finding of this study is that COVID-19-associated PE preserved the classical angiogenic profile of preeclampsia, while showing additional endothelial and inflammatory biomarker alterations.

Similar patterns were observed for several analyzed markers, including EDN1, sVCAM1, TNF-α, IL6, FASL, MMP3, PlGF and the sFlt-1/PlGF ratio, in preeclampsia cases with and without SARS-CoV-2 infection. This suggests that PE associated with SARS-CoV-2 infection shares key biological features with classical PE. The PE group associated with SARS-CoV-2 also showed additional endothelial alterations, particularly involving vWF, sVCAM1, and sICAM1 after FDR correction. However, these findings do not demonstrate that endothelial dysfunction causes PE. Rather, they suggest that PE associated with SARS-CoV-2 infection is accompanied by a distinct endothelial biomarker profile. The higher prevalence of first-trimester PE risk factors among women who later developed PE following SARS-CoV-2 infection suggests that PE associated with SARS-CoV-2 may occur preferentially in women with pre-existing susceptibility, although causality cannot be established.

Mendoza et al. [15] described a preeclampsia-like syndrome as a clinical condition resembling preeclampsia, but without the elevation of angiogenic markers typically observed in classical preeclampsia cases. Our COVID-PE cases developed preeclampsia with sFLT1/PlGF levels that tended to be even higher than those in the non-COVID-PE cases. This difference may be related to the severity of maternal COVID-19 disease or the local clinical approach with respect to PE. It has been reported that in both mild and severe COVID-19 disease, PlGF levels in maternal blood were slightly lower compared to controls [14]. As sFLT1 levels were hardly increased, the resulting sFLT1/PlGF ratios were not significantly increased in pregnant COVID-19 patients. Our results are consistent with these findings, as the sFlt-1/PlGF ratio was not increased in COVID-19 patients without preeclampsia, whereas both preeclampsia groups showed the expected antiangiogenic profile. Taken together, these findings suggest that SARS-CoV-2 infection alone was not associated with the classical angiogenic imbalance of preeclampsia, while COVID-19-associated PE preserved the angiogenic signature of classical PE.

A previous study by Palomo et al. [14] focused on the difference in circulating biomarkers between mothers suffering from either COVID-19 disease or from PE, describing alterations in most circulating biomarkers compared to non-diseased controls, although with distinctive profiles for either PE or COVID-19 disease. They reported that some biomarkers were more specifically associated with PE, including sFlt-1, whereas severe COVID-19 was associated with higher VCAM-1 and relatively lower PlGF levels. Both conditions were associated with increased VWF according to our data. In our study, COVID-PE cases also showed additional endothelial alterations, particularly involving vWF and sICAM-1 after FDR correction. These findings suggest that COVID-19-associated PE may be accompanied by a distinct endothelial biomarker profile, although causality cannot be established.

Regarding inflammatory mechanisms, it has been suggested by Papageorghiou et al. [4] that the potential mechanism linking COVID-19 and preeclampsia involves the systemic inflammatory response induced by the SARS-CoV-2 infection. In our cohort, the inflammatory biomarker profile was heterogeneous. TNF-α, IL6, and FASL did not show significant pairwise differences after FDR correction. IL8 remained increased in both PE groups compared with preCOVID-NoPE controls after FDR correction, although levels were lower in COVID-PE than in preCOVID-PE. CXCL10 levels were highest in the COVID-PE group and remained significantly increased compared with preCOVID-NoPE controls after FDR correction. These findings suggest inflammatory differences in COVID-19-associated PE, but they do not establish a generalized inflammatory mechanism or a causal pathway.

Limitations of this study should be noted. Firstly, we included cases of preeclampsia only during the initial months of the pandemic, when the women were unvaccinated and herd immunity had not yet developed, which may not reflect the current situation. However, the selected cohort provides a well-defined sample to address questions about the pathophysiological mechanisms involved. Secondly, the small sample size is an important limitation of this study, as it limits statistical power, increases the uncertainty of the estimates, and may affect the stability of medians and interquartile ranges. This limitation reflects the highly specific clinical design of the study and the difficulty of identifying well-characterized patients across four narrowly defined groups during the first year of the pandemic. Therefore, the findings should be interpreted as exploratory and hypothesis generating, and require confirmation in larger prospective cohorts. Third, although groups were selected to be comparable in terms of gestational age at blood sampling, residual confounding related to sampling time and other clinical variables cannot be excluded. Due to the limited sample size, extensive multivariable adjustment was not feasible without a substantial risk of overfitting. In addition, we tested only a very restricted number of markers. Additional markers and either functional assays or detection of endothelial damage in placentas from COVID-19 patients may further strengthen our hypothesis. The main strength of this study is the recruitment of well-characterized clinical groups, including pregnancies with PE, COVID-19, both conditions, or neither condition, with comparable gestational age at blood sampling. Overall, our results provide exploratory insights into the biomarker profile associated with COVID-19-associated PE.

In summary, PE associated with SARS-CoV-2 preserved the classical angiogenic signature of preeclampsia, characterized by increased sFlt-1, reduced PlGF, and an elevated sFlt-1/PlGF ratio, while also showing additional endothelial and inflammatory biomarker alterations. While these exploratory findings suggest that there is a distinct biomarker profile associated with PE in women with pre-existing susceptibility to the condition, they do not establish a causal relationship. The elevated levels of certain endothelial and inflammatory markers, such as CXCL10 and vWF, observed in the PE group with a concurrent SARS-CoV-2 infection are consistent with the involvement of maternal endothelial activation in the development of PE in the context of SARS-CoV-2 infection. Preeclampsia is commonly described as a disease of placental origin; however, placental transcriptomic studies have highlighted that PE is a heterogeneous syndrome with potentially different underlying biological processes [16]. Therefore, although our findings suggest a possible maternal endothelial contribution to hypertensive disorders of pregnancy associated with SARS-CoV-2, placental involvement cannot be excluded. Further research is needed in the form of larger prospective studies including longitudinal sampling, placental biomarkers, and placental tissue analysis to confirm these findings and clarify the relative contributions of the maternal and placental factors to PE associated with SARS-CoV-2 infection.

## 4. Materials and Methods

A case–control study was performed at the University Hospital Lozano Blesa (Zaragoza, Spain). Controls were defined as women with uneventful pregnancies. The control group was subdivided into women who delivered before the COVID-19 pandemic (preCOVID-NoPE group) (*n* = 10) and women who tested positive for SARS-CoV-2 during pregnancy (COVID-NoPE group) (*n* = 10). Cases were defined as women diagnosed with preeclampsia. At the same time, cases were subdivided into women who were diagnosed and delivered before the COVID-19 pandemic (preCOVID-PE group) (*n* = 10) and women who tested positive for SARS-CoV-2 during pregnancy (COVID-PE group) (*n* = 10). Controls were matched by gestational age at the time of diagnosis of preeclampsia of cases. All women included in the preCOVID-NoPE group and preCOVID-PE group delivered before 2020. All women in the COVID-NoPE and COVID-PE groups were diagnosed with SARS-CoV-2 during the first year of the pandemic, when the wildtype SARS-CoV-2 variant was predominant, and vaccines were not yet available.

All samples were obtained at HCU Lozano Blesa of Zaragoza. Preeclampsia cases (preCOVID-PE group and COVID-PE group) maternal blood samples were obtained by venipuncture onset of symptoms and before starting any treatment. Control (preCOVID-NoPE group and COVID-NoPE group) maternal blood samples were obtained around week 35 of gestation. Once blood is collected and coagulated, it is centrifuged for 10 min at 3.500× *g*; serum was aliquoted and stored at −80 °C until used. The approximate storage duration ranged from 3 to 18 months, depending on the date of sample collection. All samples were stored under the same conditions and underwent no repeated freeze–thaw cycles before biomarker measurement. Different biomarkers have been investigated in order to study endothelial damage, inflammation or angiogenesis in the study population. The following endothelial damage markers were analyzed: von Willebrand factor (vWF), endotelin 1 (EDN1), matrix metalloproteinase-3 (MMP3), soluble vascular cell adhesion molecule 1 (sVCAM1), soluble intercellular adhesion molecule-1 (sICAM1); inflammation markers: C-X-C motif chemokine ligand 10 (CXCL10), interleukin 8 (IL8), fas ligand (FASL), interleukin 6 (IL6), tumor necrosis factor (TNF-α); and modulators of angiogenesis: soluble fms-like tyrosine kinase-1 (sFLT1) and placental growth factor (PlGF). Levels of several cytokines were determined using Luminex technology [17] using standard procedures with modifications [18]. In brief, Luminex assay was run according to manufacturer’s instructions in duplicate using 25 µL of plasma, using a custom human cytokine panel (LXSAHM Discovery Assay, R&D Systems, Minneapolis, MN, USA) to assay levels of the following proteins: TNFα, IL6, CXCL10, sVCAM1, sICAM1, VWF and MMP3. Assay plates were measured using Luminex 200 equipment (Luminex Corporation, Austin, TX, USA), acquiring a minimum of 50 beads per analytes, and data were analyzed with xPONENT 3.1 software. Levels of IL8 and EDN1 were determined by cytokine-specific ELISA assays. sFlt-1 and PlGF concentrations were measured using the Elecsys^®^ sFlt-1 and PlGF immunoassay (551,275 and 563,238 Reference assay), run on Roche Cobas e801 analyzer (Roche Diagnostics, Basel, Switzerland).

The diagnosis of SARS-CoV-2 infection was confirmed through a positive RT-PCR test for SARS-CoV-2 from nasopharyngeal swabs. At that time, following the manufacturer’s recommendations for nasopharyngeal samples, cycle threshold (CT) values below 37 were considered positive. Small for gestational age (SGA) was defined as birthweight below the 10th centile according to local standards [19]. Hypertensive disorders of pregnancy (HDP) were divided into 4 categories, defined according to the criteria proposed by the International Society for the Study of Hypertension in Pregnancy [20]: preeclampsia (PE), chronic hypertension, chronic hypertension with superimposed PE and gestational hypertension. First trimester risk of early onset preeclampsia was retrospectively calculated according to maternal characteristics, obstetric history, maternal blood pressure, maternal serum pregnancy-associated plasma protein-A, and uterine artery pulsatility index, using SsdwLab6 version 6.2 package (SBP Soft 2007 S.L.) [21].

Clinical characteristics, laboratory results, and maternal and neonatal outcomes were collected from medical records. All patients signed the informed consent form and their data were pseudo-anonymized. The study has been approved by the Research Ethics Committee of Aragon (CEICA) (PI 19/227, C.I. PI21/155, and C.I. PI22/052). The project has been conducted in accordance with the principles emanating from the Declaration of Helsinki and according to current legal regulations (Fortaleza, Brazil, October 2013).

Statistical analysis was performed using SPSS version 22.0 (IBM Corp., Armonk, NY, USA) and jamovi version 2.6.44 (The jamovi project, Sydney, Australia) with the R module for false discovery rate correction. Categorical variables were summarized as frequencies and percentages, and continuous variables as median (interquartile range, IQR) or mean ± standard deviation (SD) when data followed a normal distribution. Normality was assessed using the Shapiro–Wilk test. Given the small sample size (N = 10 per group), non-parametric methods were primarily applied. Global comparisons among the four groups were performed using the Kruskal–Wallis test. Post hoc pairwise comparisons were performed using non-parametric tests. To account for multiple testing, pairwise *p*-values were adjusted using the Benjamini–Hochberg false discovery rate (FDR) procedure. Both unadjusted and FDR-adjusted *p*-values are reported, with FDR-adjusted *p*-values provided in Supplementary Table S1.

## Figures and Tables

**Table 1:** Demographic and clinical data in case–control study.

	preCOVID-NoPE Group (*n* = 10)	preCOVID-PE Group (*n* = 10)	COVID-NoPE Group (*n* = 10)	COVID-PE Group (*n* = 10)	*p*	preCOVID-NoPE Group Versus			COVID-NoPE Group Versus COVID-PE Group	preCOVID-PE Group Versus COVID-PE Group
						preCOVID-PE Group	preCOVID-PE Group	preCOVID-PE Group		
**Demographic maternal data**										
Maternal age (IQR), years	29.5 (29.0–31.5)	32.0 (27.25–41.25)	30.5 (28.0–34)	34.0 (28.5–35.75)	0.494	0.255	0.501	0.497	0.612	1
Maternal weight (IQR), kg	66.0 (56.5–74.0)	75.0 (67.0–88.0)	68.0 (54.25–70.75)	72.0 (66.25–75.75)	0.568	0.071	0.986	0.553	0.156	0.585
Gestational age at blood sampling (IQR), days	253 (252–254)	248 (243–252)	251 (244–254)	250 (241–263)	0.409	0.230	0.734	0.874	0.996	0.952
BMI > 30, %	1 (10)	4 (40)	2 (20)	2 (20)	0.268	0.121	0.531	0.531	1	0.329
Caucasian, %	6 (60)	9 (90)	2 (20)	4 (40)	**0.013**	0.121	0.068	0.371	0.329	**0.019**
Nulliparous, %	3 (30)	7 (70)	4 (40)	6 (60)	0.261	0.074	0.639	0.178	0.371	0.639
ATHc, %	0 (0)	3 (30)	0 (0)	0 (0)	0.021	0.060	1	1	1	0.06
DM, %	0 (0)	1 (10)	1 (10)	0 (0)	0.551	0.331	0.331	1	0.331	0.331
Risk PE > 100	0 (00)	4 (40)	0 (0) *	4 (44) **	**0.026**	**0.025**	1	**0.018**	**0.042**	0.845
Risk PE > 250	0 (00)	5 (62.5) *	1 (12.5) *	6 (66.6) **	**0.003**	**0.003**	0.250	**0.002**	**0.024**	0.858
**Description of SARS-CoV-2**										
Trimester of SARS-CoV-2 infection First, % Second, % Third, %	N/A	N/A	1 (10%) 5 (50%) 4 (40%)	1 (10%) 5 (50%) 4 (40%)	N/A	N/A	N/A	N/A	1	N/A
COVID-19 symptoms No Mild Severe	N/A	N/A	5 (50%) 5 (50%) 0 (0%)	4 (40%) 5 (50%) 1(10%)	N/A	N/A	N/A	N/A	0.341	N/A
**Maternal and neonatal outcome at delivery**										
Gestational age at birth (IQR), days	277.5 (274.5–280.25)	260.5 (247.0–262.75)	281.0 (275.75–285.25)	263.5 (259.25–269.25)	**<0.001**	**<0.001**	0.888	**<0.001**	0.203	**<0.001**
Preterm birth (<37 wk), %	0 (0)	3 (30%)	0 (0)	2 (20)	0.104	0.060	1	0.136	0.136	0.606
Birth weight (IQR), g	3427.5 (3348.7–3571.2)	2740.0 (2502.5–2857.5)	3147.5 (2970–3442.5)	2775.0 (2423.7–3123.7)	**<0.001**	**<0.001**	0309	**<0.001**	0.636	**0.020**
Induced labor, %	1(10)	5 (50)	3 (30)	8 (80)	**0.012**	0.051	0.624	**0.002**	0.025	0.160
ICU admission, %	0 (0)	1 (10)	1 (10)	4 (40)	0.07	0.305	0.305	**0.025**	0.121	0.121
Ph < 7.1, %	0 (0)	0 (0)	0 (0)	2 (20)	0.099	1	1	0.178	0.136	0.136

IQR: interquartile range; BMI: body mass index; ATHc: arterial hypertension chronic; DM: pregestational diabetes mellitus: Risk PE: preeclampsia risk was defined as values above >1/100 or >1/250; ICU: intensive care unit; *: *n* = 8; **: *n* = 9; N/A: not applicable; Bold numerical values indicate statistically significant differences (*p* < 0.05).

**Table 2:** Results of the biomarkers in the four study groups.

Biomarkers	preCOVID-NoPE Group (*n* = 10)	preCOVID-PE Group (*n* = 10)	COVID-NoPE Group (*n* = 10)	COVID-PE Group (*n* = 10)	*p* Overall	preCOVID-NoPE Group Versus			COVID-NoPE Group Versus COVID-PE Group	preCOVID-PE Group Versus COVID-PE Group
						preCOVID-PE Group	COVID-NoPE Group	COVID-PE Group		
**Angiogenic markers**										
**sFlt-1 (pg/mL)**	2666 (1929.5–3198.2)	5449 (3403.25–9795.25)	2346.5 (1674.7–3524.5)	15,189.0 (2209.7–24,045.0)	**<0.001**	**0.002**	0.853	**<0.001**	**<0.001**	**0.07**
**PlGF (pg/mL)**	562 (290.7–1039.0)	83.5 (55.5–139.1)	474.3 (192.8–497.1)	133.3 (90.5–246.0)	**<0.001**	**<0.001**	0.436	**0.01**	**0.005**	0.123
**sFlt-1/PIGF**	3.70 (2.2–11.0)	86.3 (50.0–140.4)	9.4 (3.4–79.3)	98.7 (72.1–176.4)	**<0.001**	**<0.001**	0.218	**<0.001**	**<0.001**	0.353
**Endothelial damage markers**										
**vWF** **(pg/mL)**	19,937 (16,849–24,742)	25,527 (12,775–35,545)	29,739 (21,468–41,641)	39,281 (30,420–61,851)	**0.006**	0.200	0.036	**0.001**	0.166	**0.013**
**EDN1** **(pg/mL)**	0.67 (0.54–0.78)	1.37 (1.20–1.76)	0.77 (0.65–0.86)	1.17 (0.72–1.48)	**<0.001**	**<0.001**	0.421	0.009	0.029	0.393
**MMP3** **(pg/mL)**	12,045 (6657–20,342)	8216 (6554–14,091)	9892 (6857–11,303)	9700 (8403–13,122)	0.353	0.311	0.158	0.295	0.449	0.927
**sVCAM1** **(pg/mL)**	413,071 (343,749–577,697)	1,181,419 (740,386–1,694,646)	502,797 (414,985–593,702)	1,124,991 (787,728–2,083,843)	**0.030**	**0.002**	0.406	**0.001**	**0.002**	0.912
**sICAM1** **(pg/mL)**	677,937 (569,677–1,401,624)	719,467 (547,564–1,117,763)	456,887 (343,250–1,086,756)	1,093,270 (648,637–2,053,676)	0.141	0.971	0.123	0.481	**0.002**	0.190
**Inflammation markers**										
**CXCL10** **(pg/mL)**	25.05 (16.34–32.77)	30.67 (24.87–52.74)	38.03 (24.13–43.74)	55.03 (44.19–62.64)	0.386	0.089	0.063	0.001	**0.043**	0.0892
**IL8** **(pg/mL)**	9.03 (6.45–16.87)	112.7 (23.03–136.2)	10.53 (7.643–18.62)	27.2 (23.12–39.20)	**<0.001**	**0.005**	0.504	**<0.001**	**0.025**	**0.006**
**FASL** **(pg/mL)**	34.07 (28.44–51.29)	29.74 (17.82–33.78)	31.28 (24.75–34.06)	32.58 (25.50–39.97)	0.131	0.048	0.161	0.409	0.515	0.151
**IL6** **(pg/mL)**	7.98 (5.81–8.84)	8.48 (6.89–13.53)	6.48 (5.50–7.61)	8.56 (7.02–10.30)	0.245	0.197	0.581	0.256	**0.045**	0.900
**TNF-α** **(pg/mL)**	37.86 (24.24–49.88)	25.72 (19.49–42.70)	21.38 (17.14–32.03)	28.83 (25.04–59.06)	0.200	0.346	0.094	0.734	0.067	0.232

Data are expressed as median (interquartile range); Global comparisons among the four groups were performed using the Kruskal–Wallis test; Pairwise comparisons are shown as unadjusted *p*-values. FDR-adjusted *p*-values for pairwise comparisons are provided in Supplementary Table S1; Bold numerical values indicate statistically significant differences (*p* < 0.05). Soluble fms-like tyrosine kinase-1 (sFLT1); placental growth factor (PlGF); von Willebrand factor (vWF); endothelin 1 (EDN1); matrix metalloproteinase-3 (MMP3); soluble vascular cell adhesion molecule 1 (sVCAM1); soluble intercellular adhesion molecule-1 (sICAM1); C-X-C motif chemokine ligand 10 (CXCL10); interleukin 8 (IL8); fas ligand (FASL); interleukin 6 (IL6); tumor necrosis factor (TNF-α).

## Data Availability

The data that support the findings of this study are not publicly available due to privacy and ethical restrictions but are available from the corresponding author upon reasonable request.

## Supplementary Materials

The following supporting information can be downloaded at: https://www.mdpi.com/article/10.3390/ijms27135891/s1.

## Abbreviations

The following abbreviations are used in this manuscript:

COVID-19	Coronavirus Disease 2019
PE	Preeclampsia
HDP	Hypertensive Disorders of Pregnancy
SARS-CoV-2	Severe Acute Respiratory Syndrome Coronavirus 2
ED	Endothelial Damage
sFlt-1	Soluble fms-like Tyrosine Kinase-1
PlGF	Placental Growth Factor
sFlt-1/PlGF	Soluble fms-like Tyrosine Kinase-1/Placental Growth Factor Ratio
sCAMs	Soluble Cell Adhesion Molecules
sVCAM1	Soluble Vascular Cell Adhesion Molecule 1
sICAM1	Soluble Intercellular Adhesion Molecule 1
VWF (or vWF)	von Willebrand Factor
EDN1	Endothelin 1
MMP3	Matrix Metalloproteinase 3
CXCL10	C-X-C Motif Chemokine Ligand 10
IL6	Interleukin 6
IL8	Interleukin 8
TNF-α	Tumor Necrosis Factor alpha
FASL	Fas Ligand
RT-PCR	Reverse Transcription Polymerase Chain Reaction
CT	Cycle Threshold
SGA	Small for Gestational Age
IQR	Interquartile Range
SD	Standard Deviation
SPSS	Statistical Package for the Social Sciences
ELISA	Enzyme-Linked Immunosorbent Assay
