# Supplementary material for: SARS-CoV-2 Infection Exacerbates Hypertensive Disorders in Pregnancy Through Vascular and Immune Pathways
Source: Int J Mol Sci. 2026 Jun 30;27(13):5891. doi: 10.3390/ijms27135891 (PMC13361972; doi:10.3390/ijms27135891)
Supplement: Supplementary file 1 [file ijms-27-05891-s001.zip › ijms-4363240-supplementary.pdf]

Biomarker	Comparison	Unadjusted p-value	FDR-adjusted p-value
sFlt-1	preCOVID-NoPE_vs_preCOVID-PE	0.002	0.009
sFlt-1	preCOVID-NoPE_vs_COVID-NoPE	0.853	0.909
sFlt-1	preCOVID-NoPE_vs_COVID-PE	0.001	0.006
sFlt-1	COVID-NoPE_vs_COVID-PE	0.001	0.006
sFlt-1	preCOVID-PE_vs_COVID-PE	0.070	0.152
PlGF	preCOVID-NoPE_vs_preCOVID-PE	0.001	0.006
PlGF	preCOVID-NoPE_vs_COVID-NoPE	0.436	0.525
PlGF	preCOVID-NoPE_vs_COVID-PE	0.010	0.033
PlGF	COVID-NoPE_vs_COVID-PE	0.005	0.019
PlGF	preCOVID-PE_vs_COVID-PE	0.123	0.228
sFlt-1/PlGF	preCOVID-NoPE_vs_preCOVID-PE	0.001	0.006
sFlt-1/PlGF	preCOVID-NoPE_vs_COVID-NoPE	0.218	0.330
sFlt-1/PlGF	preCOVID-NoPE_vs_COVID-PE	0.001	0.006
sFlt-1/PlGF	COVID-NoPE_vs_COVID-PE	0.001	0.006
sFlt-1/PlGF	preCOVID-PE_vs_COVID-PE	0.353	0.468
vWF	preCOVID-NoPE_vs_preCOVID-PE	0.200	0.310
vWF	preCOVID-NoPE_vs_COVID-NoPE	**0.036**	**0.098**
vWF	preCOVID-NoPE_vs_COVID-PE	0.001	0.006
vWF	COVID-NoPE_vs_COVID-PE	0.166	0.277
vWF	preCOVID-PE_vs_COVID-PE	0.013	0.040
EDN1	preCOVID-NoPE_vs_preCOVID-PE	0.001	0.006
EDN1	preCOVID-NoPE_vs_COVID-NoPE	0.421	0.516
EDN1	preCOVID-NoPE_vs_COVID-PE	0.009	0.031
EDN1	COVID-NoPE_vs_COVID-PE	**0.029**	**0.082**
EDN1	preCOVID-PE_vs_COVID-PE	0.393	0.511
MMP3	preCOVID-NoPE_vs_preCOVID-PE	0.311	0.430
MMP3	preCOVID-NoPE_vs_COVID-NoPE	0.158	0.275
MMP3	preCOVID-NoPE_vs_COVID-PE	0.295	0.417
MMP3	COVID-NoPE_vs_COVID-PE	0.449	0.531
MMP3	preCOVID-PE_vs_COVID-PE	0.927	0.941
sVCAM1	preCOVID-NoPE_vs_preCOVID-PE	0.002	0.009
sVCAM1	preCOVID-NoPE_vs_COVID-NoPE	0.406	0.511
sVCAM1	preCOVID-NoPE_vs_COVID-PE	0.001	0.006
sVCAM1	COVID-NoPE_vs_COVID-PE	0.002	0.009
sVCAM1	preCOVID-PE_vs_COVID-PE	0.912	0.941

sICAM1	preCOVID-NoPE_vs_preCOVID-PE	0.971	0.971
sICAM1	preCOVID-NoPE_vs_COVID-NoPE	0.123	0.228
sICAM1	preCOVID-NoPE_vs_COVID-PE	0.481	0.558
sICAM1	COVID-NoPE_vs_COVID-PE	0.002	0.009
sICAM1	preCOVID-PE_vs_COVID-PE	0.190	0.309
CXCL10	preCOVID-NoPE_vs_preCOVID-PE	0.089	0.181
CXCL10	preCOVID-NoPE_vs_COVID-NoPE	0.063	0.146
CXCL10	preCOVID-NoPE_vs_COVID-PE	0.001	0.006
CXCL10	COVID-NoPE_vs_COVID-PE	**0.043**	**0.112**
CXCL10	preCOVID-PE_vs_COVID-PE	0.089	0.181
IL8	preCOVID-NoPE_vs_preCOVID-PE	0.005	0.019
IL8	preCOVID-NoPE_vs_COVID-NoPE	0.504	0.575
IL8	preCOVID-NoPE_vs_COVID-PE	0.001	0.006
IL8	COVID-NoPE_vs_COVID-PE	**0.025**	**0.074**
IL8	preCOVID-PE_vs_COVID-PE	0.006	0.022
FASL	preCOVID-NoPE_vs_preCOVID-PE	**0.048**	**0.116**
FASL	preCOVID-NoPE_vs_COVID-NoPE	0.161	0.275
FASL	preCOVID-NoPE_vs_COVID-PE	0.409	0.511
FASL	COVID-NoPE_vs_COVID-PE	0.515	0.577
FASL	preCOVID-PE_vs_COVID-PE	0.151	0.273
IL6	preCOVID-NoPE_vs_preCOVID-PE	0.197	0.310
IL6	preCOVID-NoPE_vs_COVID-NoPE	0.581	0.640
IL6	preCOVID-NoPE_vs_COVID-PE	0.256	0.370
IL6	COVID-NoPE_vs_COVID-PE	**0.045**	**0.113**
IL6	preCOVID-PE_vs_COVID-PE	0.900	0.941
TNF-alpha	preCOVID-NoPE_vs_preCOVID-PE	0.346	0.468
TNF-alpha	preCOVID-NoPE_vs_COVID-NoPE	0.094	0.185
TNF-alpha	preCOVID-NoPE_vs_COVID-PE	0.734	0.795
TNF-alpha	COVID-NoPE_vs_COVID-PE	0.067	0.150
TNF-alpha	preCOVID-PE_vs_COVID-PE	0.232	0.343

Supplementary Table S1. Unadjusted and Benjamini-Hochberg false discovery rate (FDR)-adjusted p-values for predefined pairwise comparisons of biomarker concentrations across the four study groups. For p-values reported as $p < 0.001$ in Table 2, a value of 0.001 was used for FDR correction.Pairwise comparisons whose statistical significance changed after FDR correction are shown in bold. The FDR correction was applied across all pairwise comparisons reported in Table 2.
